# Depressed TFAM promotes acetaminophen-induced hepatotoxicity regulated by DDX3X–PGC1α–NRF2 signaling pathway

**DOI:** 10.1186/s10020-024-01017-0

**Published:** 2024-12-19

**Authors:** Sisi Chen, Yaling Cao, Zihao Fan, Ling Xu, Zhenzhen Pan, Yao Gao, Linlin Wei, Qiaoxin Wei, Yuan Tian, Xiangying Zhang, Mei Liu, Feng Ren

**Affiliations:** 1 Beijing Institute of Hepatology, Beijing Youan Hospital, Capital Medical University, No. 8, XitouTiao Road, Youwai Street, Fengtai District, Beijing, 100069 China; 2 Department of Liver Oncology, Beijing Youan Hospital, Capital Medical University, No. 8, Xitou Tiao Road, Youwai Street, Fengtai District, Beijing, 100069 China; 3 The Second Department of Liver Disease Center, Beijing Youan Hospital, Capital Medical University, Beijing, 100069 China

**Keywords:** Acetaminophen, Acute liver injury, Mitochondrial transcription factor A, Respiratory factor 2, Peroxisome proliferator-activated receptor γ-coactivator-1α

## Abstract

**Background:**

Acetaminophen (APAP)-induced acute liver injury (AILI) is the most prevalent cause of acute liver failure and mitochondrial dysfunction plays a dominant role in the pathogenesis of AILI. Mitochondrial transcription factor A (TFAM) is an important marker for maintaining mitochondrial functional homeostasis, but its functions in AILI are unclear. This study aimed to investigate the function of TFAM and its regulatory molecular mechanism in the progression of AILI.

**Methods:**

The roles of TFAM and DEAD (Asp-Glu-Ala-Asp) box polypeptide 3 X-linked (DDX3X) in AILI were determined with TFAM overexpression and DDX3X knockdown, respectively.

**Results:**

TFAM expression was suppressed in AILI patients. TFAM overexpression alleviated liver necrosis and mitochondrial dysfunction. Treatment of the AILI mice model with *N*-acetylcysteine (NAC), a drug used to treat APAP overdose, resulted in significant TFAM activation. In vivo experiments confirmed that TFAM expression was negatively regulated by DDX3X. Mechanistic studies showed that nuclear respiratory factor 2 (NRF-2), a key regulator of TFAM, was selectively activated after DDX3X knockdown via activated peroxisome proliferator-activated receptor γ coactivator 1 (PGC-1α), in vivo and in vitro.

**Conclusions:**

This study demonstrates that depressed hepatic TFAM plays a key role in the pathogenesis of AILI, which is regulated by the DDX3X–PGC1α–NRF2 signaling pathway.

**Supplementary Information:**

The online version contains supplementary material available at 10.1186/s10020-024-01017-0.

## Introduction

Acute liver failure (ALF) is defined as massive damage to liver cells in a short period of time due to various pathogenic factors, resulting in short-term failure of liver function. ALF is a rapidly progressive, complex disease with a high mortality rate (Vasques et al. 2022). There has been an increase in drug-induced ALF in the past few decades, especially due to acetaminophen (APAP)-induced liver injury (AILI) (Yan et al. 2018). APAP is a widely used analgesic and antipyretic drug with excellent therapeutic effects at the recommended doses, but unfortunately, APAP is also a dose-related toxin that can produce severe hepatotoxicity if used in excess (Lee 2017). In the United States and many European countries, nearly half of all drug-related liver injuries are caused by APAP (Shan et al. 2018). Although AILI is a great clinical challenge, its pathogenic mechanisms are not well understood and need to be studied in depth.

Excess APAP is metabolized by cytochrome P450 enzymes, primarily cytochrome P450 2E1 (CYP2E1) and CYP1A2, to form the reactive metabolite, *N*-acetyl-*p*-benzoquinone imine (NAPQI) (Lee 2017). The overproduction of NAPQI depletes glutathione (GSH) in the liver and mitochondria, while the remaining NAPQI forms covalent cellular protein associations, forming an APAP protein adduct (APAP-AD) and leading to mitochondrial damage and hepatocyte necrosis (Chao et al. 2018).The molecular mechanisms underlying the development of AILI are extremely complex and involve multiple processes such as oxidative stress, mitochondrial dysfunction, endoplasmic reticulum stress, microcirculatory disorders, and compensatory liver repair and regeneration(Bunchorntavakul and Reddy 2018; Woolbright and Jaeschke 2017). Among them mitochondrial dysfunction is considered to be a key event in AILI (Ramachandran and Jaeschke 2018).

Mitochondrial transcription factor A (TFAM) is a nuclear-encoded factor in mitochondria and a key regulator of the replication, transcription, and stabilization of mitochondrial DNA (mtDNA), promoting the expression of mitochondria-related proteins and contributing to mitochondrial biogenesis (Barshad et al. 2018). In a highly regarded publication, TFAM was described as a “master regulator of mitochondrial biogenesis” (Nisoli et al. 2005). TFAM is a downstream target of key metabolic regulators such as peroxisome proliferator-activated receptor γ coactivator 1 (PGC1) and nuclear respiratory factors (NRFs) (Virbasius and Scarpulla 1994; Gleyzer et al. 2005). It has been shown that cell type-specific TFAM-KO mice exhibit deleterious effects in cardiac myocytes (Kunkel et al. 2016), pancreatic beta cells (Li et al. 2021) and skeletal muscle (Theilen et al. 2017). During hepatic cholestasis, the damage to hepatic mtDNA is mainly due to the deletion of TFAM, which protects mtDNA against the toxic effects of bile acids on hepatocytes when overexpressed (Kang et al. 2016). In addition, it has been shown that TFAM expression in hepatocytes is significantly reduced and mitochondrial function is dysfunctional during the pathogenesis of alcoholic cirrhosis, while hepatocyte-specific TFAM overexpression prevents alcohol-induced mitochondrial dysfunction in mice (Hao et al. 2021). However, the role of TFAM in the pathogenesis of AILI has not been fully elucidated.

In this study we aimed to investigate the changes in TFAM and its role in the AILI pathogenesis and, for the first time, revealed that depressed TFAM regulated by the DEAD (Asp-Glu-Ala-Asp) box polypeptide 3 X-linked (DDX3X)–PGC1α/NRF2 signaling pathway promotes APAP-induced hepatotoxicity.

## Materials and methods

### Patients

To determine the expression of TFAM in the development and progression of AILI, serum samples were collected from 40 patients with AILI and 20 healthy controls. The clinical characteristics and details of the subjects are summarized in Supplementary Table 1. Three transplant recipients who met the Asia–Pacific Association for the Study of the Liver (APASL) AILI diagnostic criteria (Devarbhavi et al. 2021) between January 2020 and June 2021 were enrolled in this study. Liver tissue in the control group was obtained from 3 subjects who underwent hepatic resection for benign tumors. Exclusion criteria included long-term immunosuppression therapy, multiple organ failure, liver cancer, and age less than 18 years. This study adheres to the ethical principles of the 1975 Declaration of Helsinki and has been approved by the Medical Ethics Committee of Beijing YouAn Hospital. Informed consent was obtained from all patients.

### Animals

Male C57BL/6N mice between 8 and 10 weeks of age were purchased from the Beijing Weitong Lihua Experimental Animal Ltd. Co and were housed under standard conditions. All experiments were performed in strict accordance with the ethical guidelines of the Capital Medical University Animal Experimentation Committee, and the animal experimental protocols were approved by the Institutional Animal Care and Use Committee (IACUC) of Capital Medical University [AEEI-2020-009].

AILI models were constructed as follows: mice were fasted one night in advance and injected intraperitoneally with APAP (A7085, Sigma, St. Louis, MO, USA) at a concentration of 300 mg/kg. Mice were randomly divided into specific groups. Group 1, normal control (N = 6): mice were injected with an equal volume of phosphate buffer saline (PBS) according to the body weight of the mice. Group 2, APAP time-gradient processing group (N = 6 for each group): mice were injected intraperitoneally with APAP over a range of time (3, 6, 12 and 24 h). Group 3, control + LV-NC/LV-TFAM group (N = 6 for each group): LV-NC/LV-TFAM (Gene Pharma, China) (4 × 10^8^ PFU, 100µL per mouse) was injected once via tail vein one week prior to PBS treatment. Group 4, APAP + LV-NC/TFAM group (N = 6 for each group): LV-NC/LV-TFAM was injected once via tail vein one week prior to APAP treatment. Group 5, NAC group (N = 6): *N*-acetylcysteine (NAC, A7250, Sigm; 300 mg/kg, i.p.) was administered to mice 2 h after administering PBS. Group 6, APAP + NAC group (N = 6): NAC was administered to mice 2 h after administering APAP. Group 7, control + siRNA-DDX3X/NC group (N = 6 for each group): siRNA oligonucleotides against DDX3X and negative control were transfected into mice 24 h before administering PBS. Group 8, APAP + siRNA-DDX3X/NC group (N = 6 for each group): siRNA oligonucleotides against DDX3X and negative control were transfected into mice 24 h before administering APAP. Group 9, control + no-load (N = 6 for each group): no-load control viruses (Gene Pharma, China) (5 × 10^8^ PFU) at 100 µL per mouse by tail vein injection, then injection of no-load control viruses (Gene Pharma, China) (4 × 10^8^ PFU) via tail vein 4 days later and after another 7 days, PBS was injected intraperitoneally. Group 10, APAP + no-load (N = 6 for each group): no-load control viruses (5 × 10^8^ PFU) at 100 µL per mouse by tail vein injection, then injection of no-load control viruses (4 × 10^8^ PFU) via tail vein 4 days later and after another 7 days, APAP was injected intraperitoneally. Group 11, APAP + Ad-DDX3X (N = 6 for each group): DDX3X was knocked down by tail vein injection of adenovirus (Gene Pharma, China) (5 × 10^8^ PFU, 100 µL per mouse) 11 days before the administration of APAP. Group 12, APAP + Ad-DDX3X + LV-TFAM (N = 6 for each group): DDX3X was first knocked down by tail vein injection of adenovirus (5 × 10^8^ PFU, 100 µL per mouse) 11 days before the administration of APAP, and then by tail vein injection of lentivirus (Gene Pharma, China) (4 × 10^8^ PFU, 100 µL per mouse) 4 days later to knock down TFAM. After drug treatment, liver tissue and serum samples were harvested, frozen and stored in a freezer (− 80 °C) for further studies.

### Cell cultures and treatments

Primary hepatocytes were extracted from C57BL/6N mice according to the method described in the literature (Charni-Natan and Goldstein 2020), and the extracted mouse primary hepatocytes were spread on a collagen-coated cell plate. The cells cultured in Dulbecco’s modified Eagle’s medium (11960069, Gibco, Thermo Fisher Scientific, Inc., Waltham, MA, USA) containing 10% fetal bovine serum (FBS, 10099, Gibco) and 1% pen-strep (PS, 10378016, Gibco) at 37 °C and 5% CO_2_ humidified atmosphere. Cells were treated with APAP (10 mM, A7085, Sigma) for different times (0, 3, 6, 12, 24 h). To investigate the role of DDX3X in AILI, siRNA oligonucleotides against DDX3X (5 μM) and a negative control (5 μM) were transfected into primary hepatocytes cells 24 h before 12-h APAP (10 mM) stimulation. All siRNA oligonucleotides were purchased from GenePharma (Shanghai, China). A Lipofectamine 2000 kit (11668030, Invitrogen, Thermo Fisher Scientific, Inc., Waltham, MA, USA) was used to transfect specific siRNAs according to the manufacturer’s instruction.

### Liver injury assessment

Serum alanine aminotransferase (ALT) and aspartate aminotransferase (AST) were detected by an automated chemical analyzer (Olympus Company, Tokyo, Japan). Liver tissues were fixed with formaldehyde and embedded in paraffin, stained with hematoxylin and eosin (H&E), and then examined under a light microscope.

### Western blot

Proteins were extracted from primary hepatocytes or mouse liver tissue using RIPA solutions containing protease and phosphatase inhibitors. Protein quantification was done by a bicinchoninic acid (BCA) protein determination kit (23225, Thermo Fisher Scientific) according to the manufacturer’s instructions. Proteins were separated by10-12% SDS–polyacrylamide gel electrophoresis and then transferred to a PVDF membrane at 4 °C. Monoclonal antibodies against TFAM (1:1000, ab252432, Abcam, Inc, Cambs, UK), PGC-1α (1:1000, Ab54481, Abcam), jun-N-terminal kinase (JNK, 1:1000, 9252, Cell Signaling Technology, Inc, MA, USA), p-JNK (1:1000, 9255, Cell Signaling Technology), extracellular regulated protein kinase(ERK, 1:1000, 4695, Cell Signaling Technology), p-ERK (1:1000, 4370, Cell Signaling Technology), p38 (1:1000,9212, Cell Signaling Technology), p-p38 (1:1000, 4511, Cell Signaling Technology), DDX3X (1:1000, 8192, Cell Signaling Technology), NRF-2 (1:1000, ab88746, Abcam),Tubulin (1:1000, 2144, Cell Signaling Technology), Histone H3 (1:1000, 60932, Cell Signaling Technology) were diluted in TBST buffer with 5% skim milk, and then they were incubated at 4 °C overnight. The next day, the membranes were washed three times with TBST buffer. Then they were incubated with horseradish peroxidase-conjugated secondary antibody (1:2000, A-10668; G-21234, Cell Signaling Technology) for 60 min at room temperature and washed three times. Subsequently, the bands were visualized using an enhanced chemiluminescence detection kit (32209, Thermo Fisher Scientific).

### Quantitative real-time polymerase chain reaction (qRT-PCR)

FastPure® Cell/Tissue Total RNA Isolation Kit (RC112-01, Vazyme Biotech Co., Ltd., Nanjing, China) was used to extract total RNA from cells and liver tissue. The HiScript® III RT SuperMix for qPCR kit (R323, Vazyme) was then used to reverse transcribe the RNA to cDNA. The PCR reaction system was 20 µL, which included 10 μL SYBR Green (RR420A, TaKaRa Biotechnology Co., Ltd., Dalian, China), 0.4 μL forward and reverse primers, 5.2 μL enzyme-free sterile water and 4 μL cDNA. The PCR reaction conditions were as follows: 50 °C for 2 min and 95 °C for 5 min followed by 44 cycles of 95 °C for 15 s, 60 °C for 30 s, and 55 °C for 4 s. Relative levels of target mRNA expression were analyzed using the 2^−ΔΔCt^ method. List of primers were summarized in Supplementary Table 2.

### Electron microscope observation

The liver tissues loaded into the electron microscope fixative were rinsed in 1 × PBS buffer three times for 15min each. The liver tissues were treated with 1% osmium acid solution and fixed for 2 h (this process should be performed in a fume hood), and then rinsed in 1 × PBS buffer three times for 15 min each. The samples were sequentially dehydrated in 30% acetone-50% acetone-70% acetone-anhydrous acetone three times for 30 min each. The samples were then placed in anhydrous acetone: embedding agent (1:1) for 3h and anhydrous acetone: embedding agent (1:1) for overnight. The infiltrated samples were placed in embedding plates and polymerized at 45 °C for 12 h and 60 °C for 36–48 h. The samples were cut into 60–80 nm sections using an ultra-thin sectioning machine, double-stained with uranium-lead, and dried overnight. The sections were then placed under a transmission microscope to observe the mitochondrial morphology and photographed, and the images were collected and saved in a fixed folder for subsequent analysis.

### Immunofluorescence

Paraffin sections of mouse liver tissue were dewaxed and fixed in paraformaldehyde on ice for 10 min. The sections were then washed three times with PBS buffer and treated with Triton X-100 (9036-19-5, Sigma) to permeabilize the cell membranes. Blocked with 1% goat serum and 3% BSA for 1 h at 37 °C, followed by incubation with 3% BSA and a rabbit TFAM (1:500, ab252432, Abcam) overnight at 4 °C. After washing in PBS, sections were incubated with Alexa Fluor 488-conjugated goat anti-rabbit IgG antibody (1:250, B40943, Invitrogen) at 37 °C for 30 min. Cell nuclei were stained with 6-diamino-2-phenylindole (DAPI, 1 μg/mL, 4083, Abcam) for 10 min and analyzed under a Leica DM2500 fluorescence microscope.

### Glutathione (GSH) assay

Liver tissue GSH levels were measured using the reduced glutathione content assay kit (BC1175, Solarbio Life Sciences, Co., Ltd., Beijing, China). The assay was performed according to the manufacturer’s instructions.

### Reactive oxygen species (ROS) measurements

Frozen sections were rewarmed at room temperature and water was controlled to dry. A circle was drawn around the tissue with a histochemical pen, autofluorescence quench (Servicebio Biotechnology Co., Ltd., Wuhan, China) was added for 5min, and the tissue was rinsed with running water for 10min. ROS staining solution (200–664-3, Sigma) was added drop inside the circle and incubated for 30min at 37 °C dark incubator. The slides were washed 3 times in PBS solution by shaking them on a decolorization shaker for 5 min each time. DAPI staining solution (G1012, Servicebio) was added and incubated for 10 min at room temperature in the dark. The slides were washed three times in PBS solution by shaking them on a decolorization shaker for 5 min each time. Finally, the images were analyzed by a NIKON ECLIPSE C1 fluorescence microscope.

### Quantitative real-time PCR for the mtDNA content

The D-loop region within the mtDNA genome was quantitatively amplified by TaqMan PCR to analyze serum mtDNA levels in total DNA isolated from 25 to 50 µL of serum (Gentra Puregene kit, 158845, Qiagen, Dusseldorf, GER). Relative transcript levels were quantified by real-time RT-PCR using the TaqMan methodology. TaqMan probes and primers (summarized in Supplementary Table 2) were designed and validated previously (An et al. 2020).

### Nucleus-cytoplasm separation assay

Nucleus-cytoplasm separation assay was performed with the nuclear/cytosol fractionation kit (ab289882, Abcam) according to the manufacturer’s instructions.

### Statistical analysis

Statistical analysis was performed using GraphPad Prism 8.0 software and experimental data were expressed as means ± standard deviation. Statistical analysis was performed using an unpaired t-test or one-way analysis of variance (ANOVA) to evaluate the differences between groups. P-values were calculated and p-values < 0.05 were considered statistically significant.

## Results

### Changes in the expression of mtDNA and TFAM in the liver tissues and serum of AILI patients

**Fig. 1:**
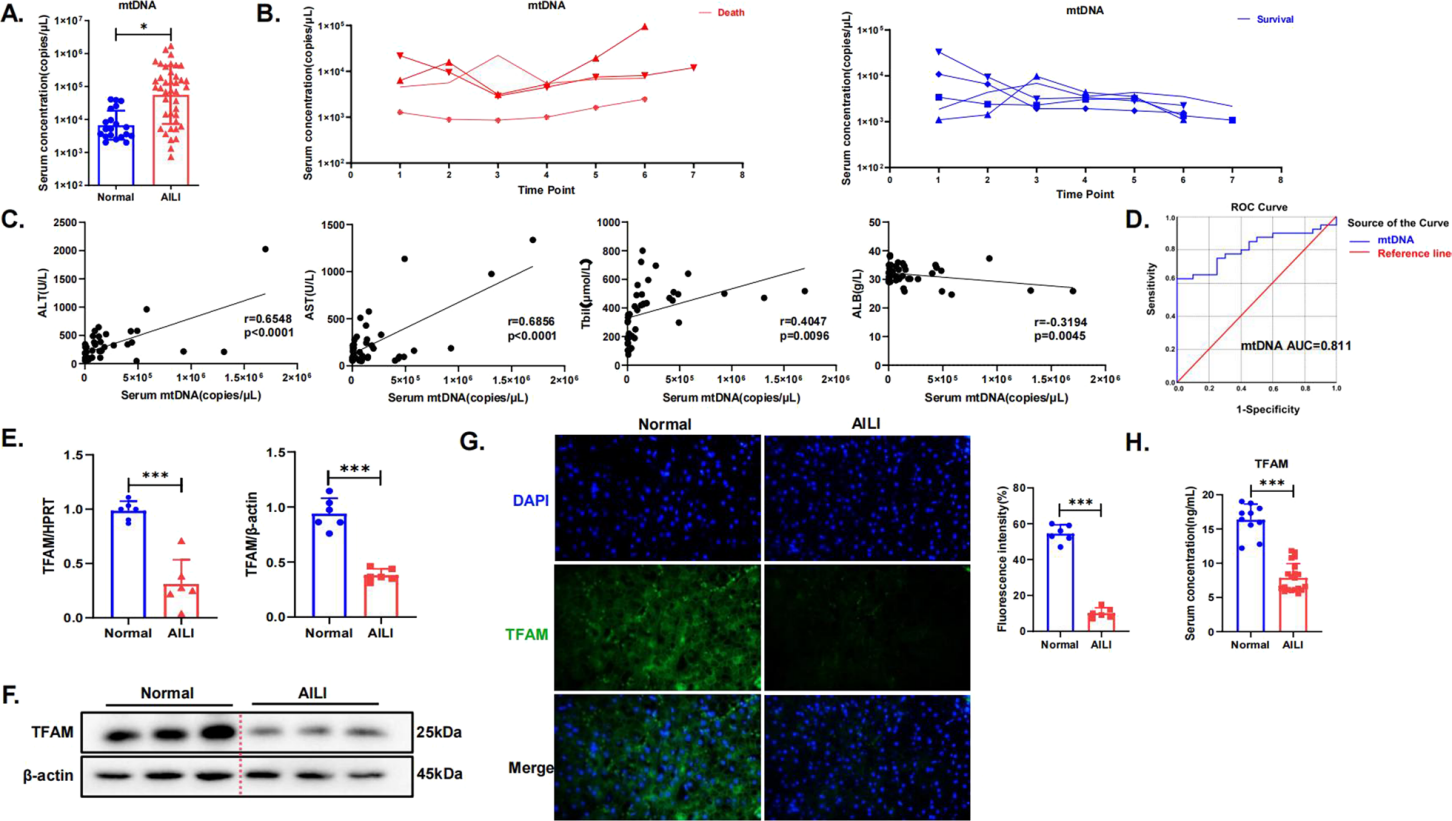
Changes in the expression of mtDNA and TFAM in the liver tissues and serum of AILI patients. **A** Serum levels of mtDNA in normal subjects and in AILI patients. **B** Changes in the serum mtDNA levels of nine AILI patients, including four who died and five who survived. **C** The correlation between serum mtDNA levels and liver injury in AILI subjects was assessed by Pearson correlation analysis, including serum ALT levels, AST levels, ALB levels and Tbil levels. **D** Diagnostic value potential of mtDNA for AILI. **E** The gene expression of TFAM in normal subjects and AILI patients was measured by quantitative real-time PCR. **F** The protein levels of TFAM in human liver tissue samples were detected by western blotting and quantitated with the ImageJ software. **G** TFAM staining of liver tissue samples (green: TFAM-positive cells; blue: DAPI; scale bar, 10 µm). **H** Serum levels of TFAM in normal subjects and in AILI patients. ^*^p < 0.05; ^**^p < 0.01; ^***^p < 0.001; ^****^p < 0.0001; ns not significant

We collected peripheral serum from 20 healthy individuals and 40 patients with AILI, and the demographic and clinical characteristics of participants in the different serum study groups are shown in Supplementary Table 1. mtDNA in the serum was examined, and we found that the level of mtDNA in the serum of patients with AILI was significantly higher than that in the normal population (Fig. 1A). Next, we explored the relationship between changes in serum mtDNA levels and different prognoses in a representative sample of AILI patients, including four nonsurvivors and five survivors. There was an overall decreasing trend in mtDNA levels in all surviving patients and an overall increasing trend in mtDNA levels in all nonsurvivors during the progression of AILI (Fig. 1B). We evaluated the possible correlation between serum mtDNA levels and liver injury in AILI patients. Pearson correlation analysis showed that serum ALT levels, AST levels, and total bilirubin (Tbil) levels were significantly positively correlated with serum mtDNA levels, while albumin (ALB) levels were significantly negatively correlated with serum mtDNA levels (Fig. 1C). Receiver operating characteristic (ROC) curves were constructed to assess whether mtDNA could be used as a diagnostic biomarker for AILI. Serum mtDNA showed high accuracy in differentiating AILI from normal subjects with an area under the ROC curve (AUROC) of 0.811 (95% CI 0.704–0.918, *p* < 0.001) (Fig. 1D). Thus, circulating mtDNA is a potential diagnostic biomarker for AILI.

Since TFAM can regulate mtDNA copy number and is closely related to the replication and stability of mtDNA, we examined the expression content of TFAM in the liver tissues of liver donors and AILI patients. The WB, PCR, and immunofluorescence results all showed significantly lower TFAM expression levels in the liver tissue of AILI patients than in that of normal subjects (Fig. 1E–G). TFAM was detected by ELISA in the serum of normal subjects and AILI patients and was significantly reduced in the serum of AILI patients (Fig. 1H). These findings suggest that in AILI, serum mtDNA levels are increased, while liver tissue and serum TFAM levels are significantly decreased.

### Expression profile of TFAM in APAP-induced mouse hepatotoxicity

**Fig. 2:**
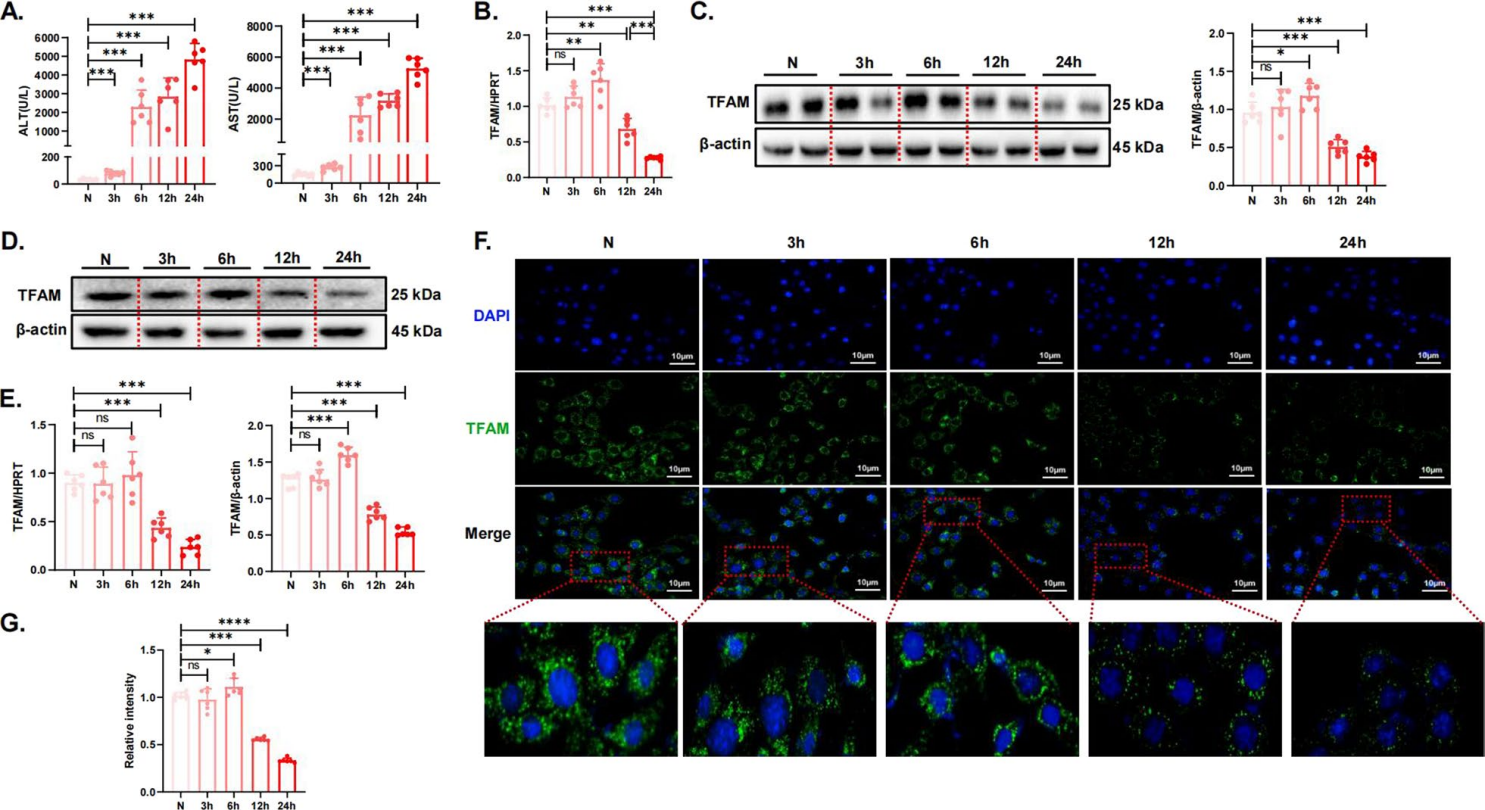
Expression profile of TFAM in APAP-induced mouse hepatotoxicity. **A** Serum AST and ALT enzyme levels. N = 6 for each group. **B** The gene expression of TFAM in mouse liver tissue samples was measured by quantitative real-time PCR. **C** The protein levels of TFAM in mouse liver tissue samples were detected by western blotting and quantitated with the ImageJ software. The blots and images are representative of three independent experiments. **D** Protein levels of TFAM in primary hepatocyte samples were detected by western blotting. The blots and images are representative of three independent experiments. **E** The gene expression of TFAM in primary hepatocytes was measured by quantitative real-time PCR. The protein levels of TFAM in primary hepatocyte samples were quantitated with the Image J software. Data from 3 independent experiments are shown as mean ± SD. **F** TFAM staining of primary hepatocyte samples (green: TFAM-positive cells; blue: DAPI; scale bar, 10 µm). **G** The quantitative immunofluorescence plots were measured with ImageJ software. Data from 3 independent experiments are shown as mean ± SD. ^*^p < 0.05; ^**^p < 0.01; ^***^p < 0.001; ^****^p < 0.0001; ns not significant

C57 mice were treated with 300 mg/kg APAP at different times to induce AILI to varying degrees, and changes in liver morphology and ALT and AST levels were evident after 6 h (Figure S1A, 2A). In addition to liver injury, time-dependent APAP treatment significantly reduced TFAM expression in the liver at 12h, which was further reduced at 24 h (Fig. 2B, C). In addition, we found that concentration gradient-dependent APAP treatment for 12 h also significantly reduced TFAM expression (Figure S1B-D). In vitro experiments, primary hepatocytes were treated with 10 mM APAP at different times. Consistent with the in vivo results, TFAM RNA and protein levels began to decrease at 12 h and further decreased at 24 h (Fig. 2D, E). These alterations were also confirmed by immunofluorescence staining (Fig. 2F, G). These results indicated that TFAM was significantly reduced during the progression of AILI.

### TFAM overexpression alleviates APAP-induced liver injury in mice

**Fig. 3:**
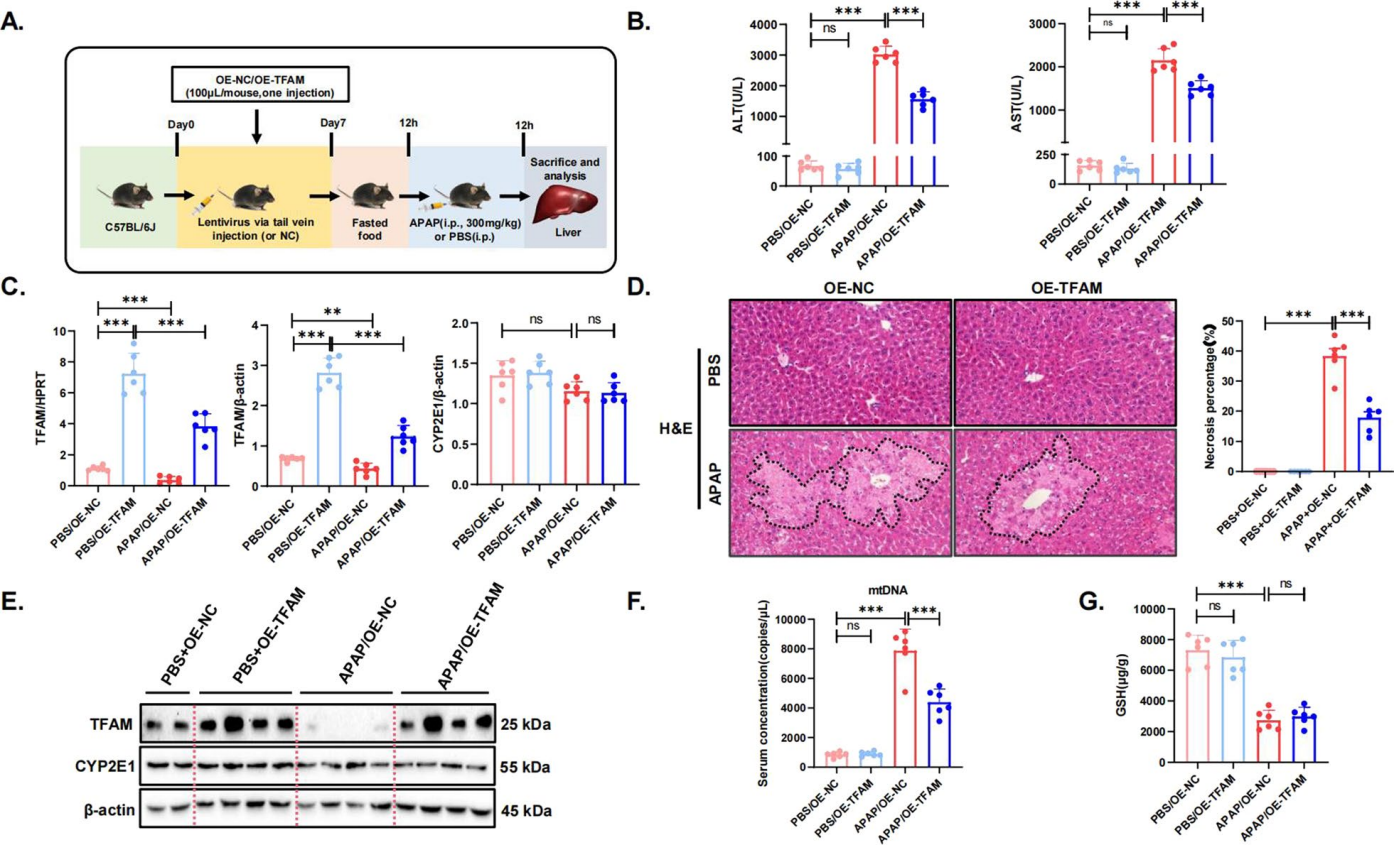
TFAM overexpression alleviates APAP-induced liver injury in mice. **A** Schematic representation of mouse model establishment and lentivirus injection. N = 4 for each group. N = 6 for each group. **B** Serum AST and ALT enzyme levels. **C** Hepatic mRNA and protein expression of TFAM and CYP2E1 in the different groups. **D** Representative images of H&E-stained liver sections. The areas surrounded by a black line and arrow are areas of liver tissue damage (magnification: 200×). Data represent the mean ratio of the necrotic area to the total area ± SD. **E** Protein levels of TFAM and CYP2E1 in mouse liver tissue samples were detected by western blotting and quantitated with the ImageJ software. The blots and images are representative of three independent experiments. **F** Serum levels of mtDNA in the different groups. **G** GSH levels in liver tissues in the different groups. ^*^p < 0.05; ^**^p < 0.01; ^***^p < 0.001; ^****^p < 0.0001; ns not significant

To determine the role of TFAM in the pathogenesis of AILI, a TFAM overexpression lentivirus was generated. The lentivirus was administered to C57 mice via tail vein injection, and 7 days later, 300 mg/kg APAP treatment was administered for 12 h. (Fig. 3A). Relative to that in the negative control group, TFAM expression was significantly higher in the OE-TFAM group (Fig. 3C, E). It is worth noting that the increases in ALT levels, AST levels, and the area of liver necrosis induced by APAP treatment were greatly reduced in the TFAM overexpression group mice compared with the negative control group mice (Fig. 3B, D). APAP treatment resulted in a significant increase in mtDNA levels in the serum of mice, while TFAM overexpression significantly decreased mtDNA levels in serum (Fig. 3F). GSH protects cells from oxidative damage by removing ROS or quenching NAPQI, which is the reactive metabolite of APAP. TFAM overexpression did not alter the levels of NAPQI and GSH in mouse liver compared with that in the negative control group, suggesting that OE-TFAM may not exert a protective effect by affecting APAP metabolism and that other regulatory mechanisms may exist (Fig. 3C, E, G). In addition, APAP-treated mice showed disturbed mitochondrial morphology in the liver, which was characterized by swollen mitochondria and broken mitochondrial cristae, while TFAM overexpression significantly improved this phenomenon and normalized the mitochondrial morphology (Figure S2A). Increased levels of total ROS were detected in the liver tissue of APAP-treated mice, and these effects were ameliorated by TFAM overexpression (Figure S2B). These results showed that TFAM overexpression alleviates liver injury and mitochondrial dysfunction caused by APAP.

It has been demonstrated that the toxicity of APAP is related to the MAPK pathway. The pathway involves a variety of key protein kinases and regulators, such as extracellular signal-regulated kinase (ERK), c-Jun N-terminal kinase (JNK), and p38 MAPK (Deng et al. 2024). We examined the expression of the MAPK signaling pathway in the TFAM overexpression animal models and showed that the phosphorylation levels of JNK and ERK (p-JNK, p-ERK) were significantly reduced in the APAP/LV-TFAM group compared to the APAP stimulation group alone, whereas there was no significant change in the phosphorylation level of p38 (Figure S3A, B). These results showed that TFAM overexpression may exert a protective effect by inhibiting the phosphorylation levels of JNK and ERK, key molecules of the MAPK signaling pathway.

Mitochondrial damage plays a key role in the development of AILI, and mitophagy is an important process to remove damaged mitochondria (Lu et al. 2023), so we examined the effect of overexpression of TFAM on mitophagy-related molecules, including four key molecules: BCL2/adenovirus E1B 19kDa interacting protein 3 (BNIP3), PTEN induced putative kinase 1 (PINK1), Parkin and FUN14 domain containing 1 (FUNDC1). PCR results showed that the expression of these four molecules was significantly down-regulated in the APAP model group, as compared with the blank control group. The PCR results showed that mitophagy was inhibited in the APAP model group compared with the blank control group, and the expression levels of BNIP3, PINK1, and Parkin were up-regulated by overexpression of TFAM, while FUNDC1 was not significantly affected (Figure S4), which suggests that TFAM may improve the function of mitochondria by affecting mitophagy and thus play a protective role.

### N-Acetylcysteine (NAC) treatment alleviates APAP-induced hepatotoxicity by upregulating TFAM expression

**Fig. 4:**
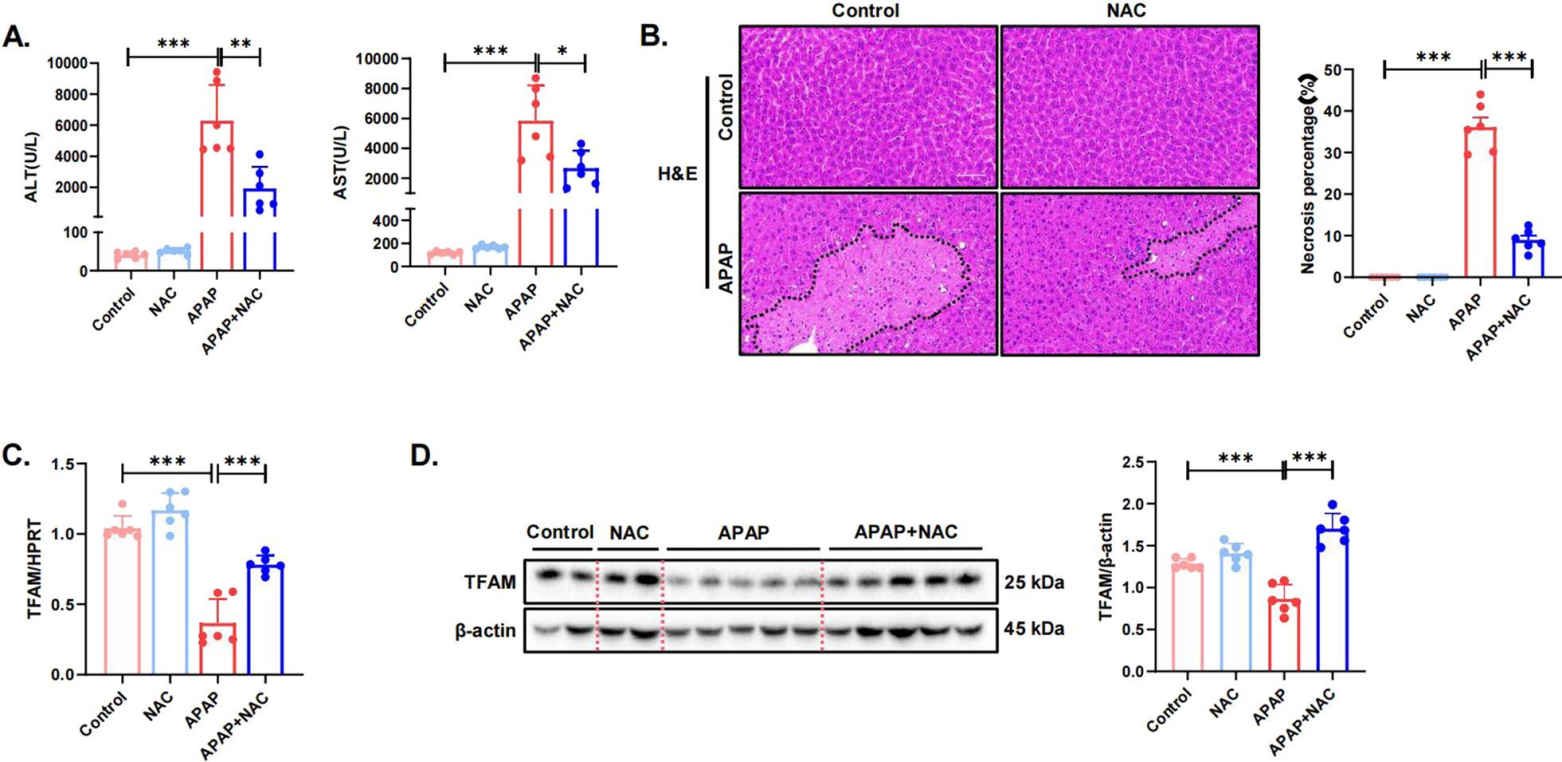
N-Acetylcysteine (NAC) treatment alleviates APAP-induced hepatotoxicity by upregulating TFAM expression. **A** Serum AST and ALT enzyme levels. N = 6 for each group. **B** Representative images of H&E-stained liver sections. The areas surrounded by a black line and arrow are areas of liver tissue damage (magnification: 200×). Data represent the mean ratio of the necrotic area to the total area ± SD. **C** The gene expression of TFAM in mouse liver tissue samples was measured by quantitative real-time PCR. **D** The protein levels of TFAM in mouse liver tissue samples were detected by western blotting and quantitated with the ImageJ software. The blots and images are representative of three independent experiments. ^*^p < 0.05, ^**^ *p* < 0.01, ^***^ p < 0.001

NAC was developed as an antidote to APAP poisoning and remains an important preventative measure to protect the liver from APAP-induced toxicity (Devarbhavi et al. 2021). APAP overdose leads to significantly decreased GSH levels, which NAC is believed to boost (Raghu et al. 2021). Therefore, we investigated whether the treatment with NAC affected the reduction in TFAM expression caused by APAP. NAC solution (300 mg/kg) or PBS solution was injected intraperitoneally 2 h after APAP administration. Compared with those in the APAP-induced liver injury group, serum levels of ALT and AST were significantly lower in the mice that were treated with NAC (Fig. 4A). While the results of H&E staining were consistent with the liver function results, the area of liver necrosis was reduced in the group that was treated with NAC (Fig. 4B). We then examined the hepatic TFAM expression in the NAC-treated group of mice and found that NAC treatment significantly alleviated the reduction in TFAM expression caused by APAP at the gene and protein levels (Fig. 4C, D). These results showed that NAC treatment significantly alleviated the acute liver injury caused by APAP and the decrease in TFAM expression.

### DDX3X promotes APAP-induced liver injury by negatively regulating TFAM in vivo

**Fig. 5:**
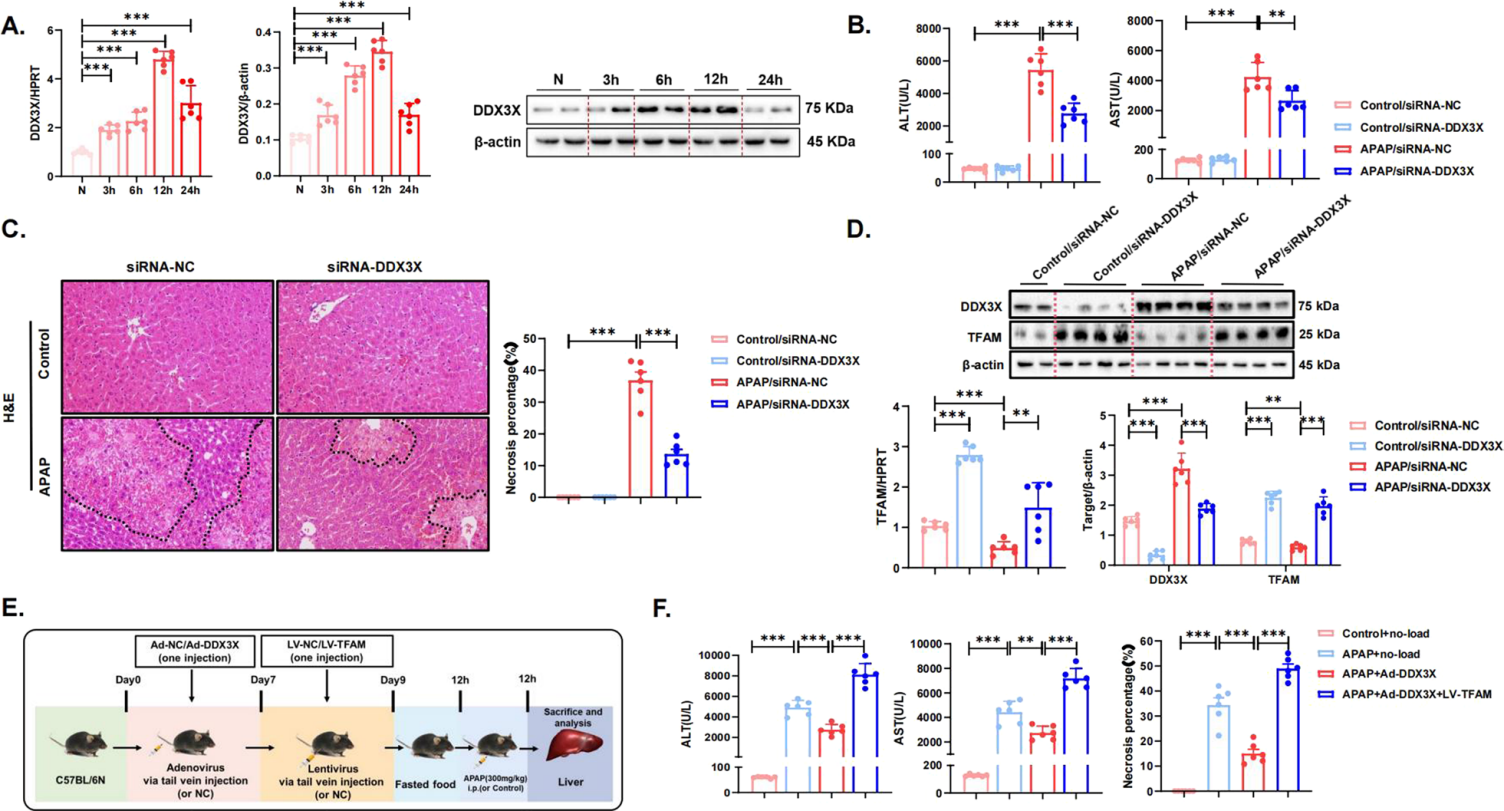
DDX3X promotes APAP-induced liver injury by negatively regulating TFAM in vivo. **A** The hepatic mRNA and protein expression of DDX3X in mouse liver tissue samples (N = 6). The blots and images are representative of three independent experiments. **B** Detection of the effect of DDX3X knockdown on serum ALT and AST in the control or APAP model group. **C** Representative images of H&E-stained liver sections. The areas surrounded by a black line and arrow are areas of liver tissue damage (magnification: 200×). Data represent the mean ratio of the necrotic area to the total area ± SD. **D** The gene expression of TFAM was measured by quantitative real-time PCR in mouse liver tissue from AILI mice with DDX3X knockdown. The protein levels of TFAM in mouse liver tissue samples of AILI mice with DDX3X knockdown were detected by western blotting and quantitated with the ImageJ software. The blots and images are representative of three independent experiments. **E** Schematic representation of mouse model establishment and virus injection. N = 6 for each group. **F** DDX3X was knocked down, followed by TFAM in the AILI model, and serum ALT and AST levels in the model group and the single-knockdown and double-knockdown intervention groups were measured. Data represent the mean ratio of the necrotic area to the total area ± SD. ^*^p < 0.05, ^**^p < 0.01, ^***^ *p* < 0.001, ^****^ *p* < 0.0001

The DDX3X gene on the X chromosome encodes an ATP-dependent, DEAD-box RNA helicase involved in RNA metabolism (Mo et al. 2021). It has been shown that targeting DDX3X inhibits mitochondrial translation, subsequently decreasing oxidative phosphorylation (OXPHOS) and increasing ROS production, causing metabolic disturbances in cancer cells and ultimately leading to cell death. This finding demonstrates that DDX3X is closely associated with mitochondrial homeostasis and functional metabolism (Fu et al. 2019). We therefore investigated whether DDX3X plays a role in the pathogenesis of AILI, which is mainly characterized by mitochondrial dysfunction, and whether there is an interaction between DDX3X and TFAM, another key molecule for maintaining mitochondrial homeostasis. Intriguingly, APAP-treated mice exhibited higher hepatic DDX3X mRNA and protein levels than controls (Fig. 5A). Cellular experiments in vivo were consistent with the in vitro results (Figure S5A, B). In addition, the mRNA and protein expression of DDX3X gradually increased with the extension of APAP treatment time, and the highest expression occurred at 12 h, after which it started to decrease.

Then we knocked down DDX3X with siRNA to investigate its effect on APAP-induced liver injury. We found that DDX3X knockdown significantly attenuated APAP-induced liver injury, and reduced serum levels of ALT and AST, and the H&E staining results indicated a reduction in the necrotic area of the liver (Fig. 5B, C). In addition, mtDNA levels in serum were significantly reduced after DDX3X knockdown (Figure S5C). To further investigate whether DDX3X interacts with TFAM, we examined the DDX3X expression levels in the liver tissues of mice overexpressing TFAM and TFAM expression levels in the liver tissues of mice with DDX3X knockdown. The results showed that TFAM overexpression did not affect the hepatic mRNA and protein levels of DDX3X (Figure S7A, B), while DDX3X knockdown caused a significant increase in the mRNA and protein levels of TFAM in the control and APAP-treated groups (Fig. 5D). Since the DDX3X gene is located on the X chromosome, in order to verify whether there is a sex-dependent characterization of it, we supplemented the data from the DDX3X knockout experiments in female mice (as opposed to male mice). The results were consistent with those observed in male mice (Figure S6A–D). Thus, DDX3X expression is increased in AILI and may exert a pro-damage effect by inhibiting TFAM expression. We then examined whether DDX3X knockdown had any effect on the GSH content of mouse liver tissues, and we found that knockdown of DDX3X did not significantly alleviate the reduction of GSH content caused by APAP stimulation, suggesting that knockdown of DDX3X may not act through the pathway of affecting APAP metabolism (Figure S7C).

To further demonstrate that DDX3X exerts a pro-injury effect by inhibiting TFAM expression during the pathogenesis of AILI, we performed a double knockdown experiment of DDX3X and TFAM, and the establishment of the specific animal model is shown in Fig. 5E. We found that liver function in APAP-treated mice was significantly better in the mice with DDX3X knockdown alone than in the mice that were injected with no-load control virus, while the liver function of the mice with DDX3X knockdown followed by TFAM knockdown deteriorated further (Fig. 5F). The HE staining results were similar to the liver function results, and the area of liver necrosis in mice with DDX3X knockdown alone was significantly reduced compared with that in null mice. In contrast, mice with further TFAM knockdown on top of DDX3X knockdown showed a large area of necrosis in the liver, which was much larger than that of mice with DDX3X knockdown alone (Fig. 5F, Figure S8A). These results suggest that TFAM knockdown eliminates the hepatoprotective effect mediated by the inhibition of DDX3X.

### DDX3X promotes APAP-induced liver injury by inhibiting the PGC1α-NRF2-TFAM signaling pathway

**Fig. 6:**
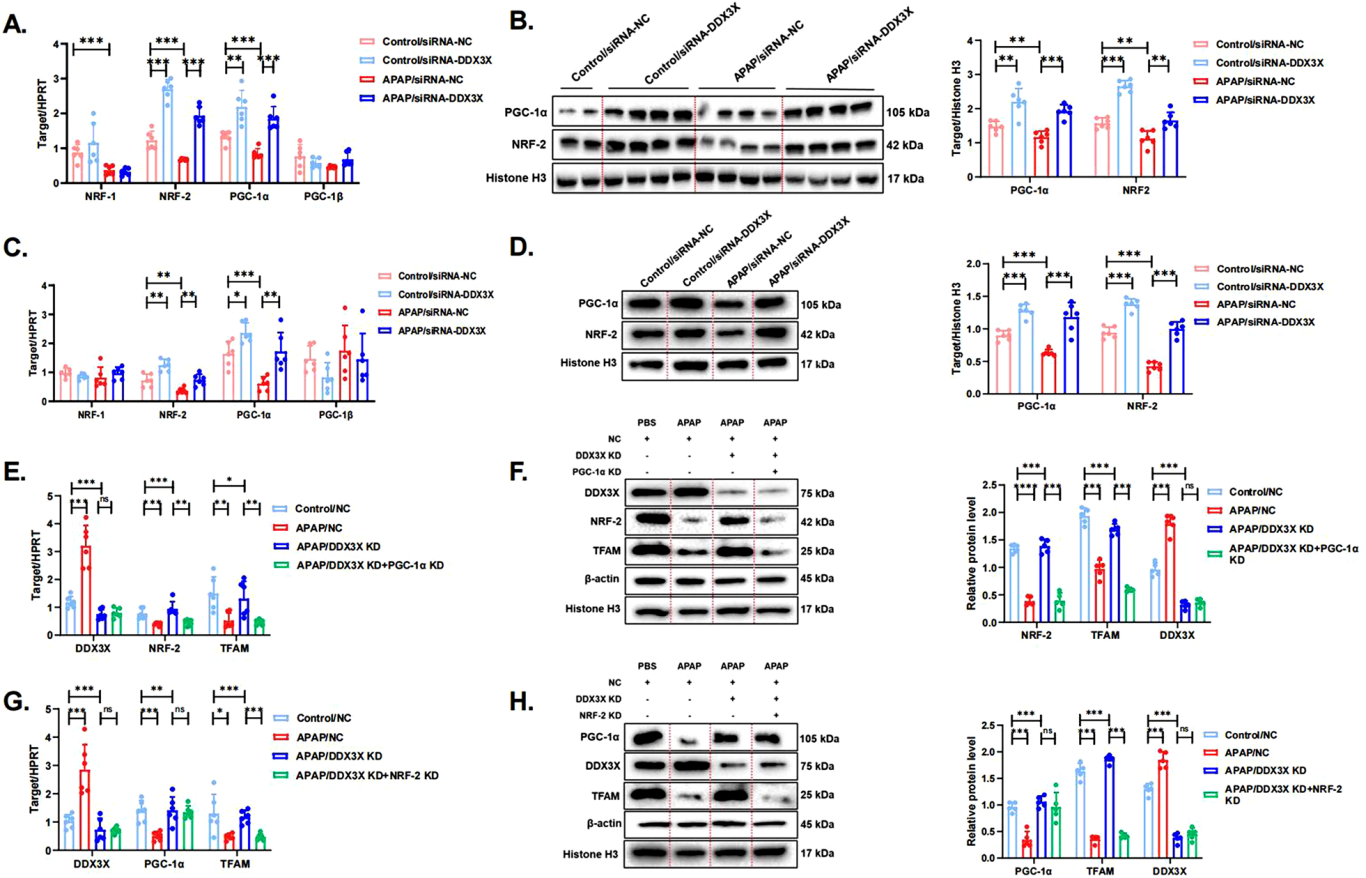
DDX3X promotes APAP-induced liver injury by inhibiting the PGC1α-NRF2-TFAM signaling pathway. **A** The gene expression of major signaling molecules associated with mitochondrial biogenesis in liver tissues of AILI mice with DDX3X knockdown were examined using quantitative real-time PCR. N = 6 for each group. The blots and images are representative of three independent experiments. **B** The nuclear protein levels of PGC-1α and NRF-2 in the liver tissue samples of AILI mice with DDX3X knockdown were detected by western blotting and quantitated with the ImageJ software. **C** The gene expression of major signaling molecules associated with mitochondrial biogenesis in APAP-stimulated cells with DDX3X knockdown was examined using quantitative real-time PCR. **D** The nuclear protein levels of PGC-1α and NRF-2 in APAP-stimulated cells with DDX3X knockdown were detected by western blotting and quantitated with the ImageJ software. Data from 3 independent experiments are shown as mean ± SD. **E** Single DDX3X knockdown or double knockdown of DDX3X and PGC-1α in the AILI cell model was performed, and the gene expression of DDX3X, NRF-2 and TFAM in the different subgroups was measured. **F** The protein levels of DDX3X, NRF-2 and TFAM in cells with single DDX3X knockdown or double knockdown of DDX3X and PGC-1α were detected by western blotting and quantitated with the ImageJ software. Data from three independent experiments are shown as mean ± SD. **G** Single DDX3X knockdown or double knockdown of DDX3X and NRF-2 in the AILI cell model was performed, and the gene expression of DDX3X, PGC-1α and TFAM in the different subgroups was measured. **H** The protein levels of DDX3X, PGC-1α and TFAM in cells with single DDX3X knockdown or double knockdown of DDX3X and NRF-2 were detected by western blotting and quantitated with the ImageJ software. Data from three independent experiments are shown as mean ± SD. ^*^p < 0.05, ^**^p < 0.01, ^***^ *p* < 0.001, ^****^ *p* < 0.0001; ns not significant

To explore the possible mechanisms by which DDX3X negatively regulates TFAM, we examined the effects of APAP and DDX3X knockdown on major signaling molecules upstream of TFAM that are associated with mitochondrial biogenesis (Gleyzer et al. 2005; Kunkel et al. 2016). As shown in Fig. 6A, the mRNA levels of NRF-2 and PGC-1α, but not NRF-1 and PGC-1β, were increased in control/siRNA-DDX3X mice compared with control/siRNA-NC mice. In addition, DDX3X knockdown reversed the APAP-induced reduction in NRF-2 and PGC-1α expression, suggesting that DDX3X may suppress TFAM expression through inhibiting NRF2/PGC-1α (Fig. 6A). The WB results and PCR results were consistent, indicating that the APAP-induced decrease in liver NRF-2 and PGC-1α protein levels was reversed by DDX3X knockdown (Fig. 6B). Then, we further validated these results in cell models. DDX3X was knocked down in primary hepatocytes with small interfering RNAs, followed by the stimulation of control and model cells with 10 mM APAP solution for 12 h. We found that DDX3X knockdown in the cells that were treated with PBS resulted in significantly increased levels of NRF-2 and PGC-1α expression (Fig. 6C). While APAP decreased NRF-2 and PGC-1α expression levels in hepatocytes, DDX3X knockdown reversed this decrease in expression levels (Fig. 6C). The WB results showed that APAP treatment resulted in reduced expression levels of nuclear protein levels of NRF-2 and PGC-1α compared to those in the control group, while DDX3X knockdown increased the nuclear protein levels of NRF-2 and PGC-1α in the control and APAP-treated model groups (Fig. 6D). These results all indicate that DDX3X negatively regulates NRF2/PGC-1α and may further suppress TFAM expression through this pathway. In addition, we also examined the expression levels of PGC-1α and NRF-2 in APAP time-gradient-stimulated cells and animal models. PCR and WB results showed that the expression levels of PGC-1α and NRF-2 were reduced from 12 h and further significantly reduced at 24 h, both in the cell and animal models (Figure S9A-D).

We then used siRNA to interfere with the expression of NRF-2 and PGC-1α (Figure S10A–D) to investigate whether these factors bidirectionally regulate DDX3X and their relationships. The results showed that knockdown of PGC-1α significantly reduced the mRNA and protein levels of TFAM, while the mRNA and nuclear protein levels of NRF-2 were also significantly reduced. However, knockdown of PGC-1α had no effect on the expression of DDX3X (Fig. 6E, F). Subsequently, we also knocked down NRF-2 and found that NRF-2 knockdown significantly reduced the mRNA and protein levels of TFAM in AILI cells, but knocking down NRF-2 had no effect on the expression of DDX3X or PGC-1α (Fig. 6G, H). In addition, the APAP-reduced reductions in hepatic PGC-1α, NRF-2 and TFAM expression levels were reversed by DDX3X deletion, whereas the DDX3X knockdown-induced increases in TFAM and NRF-2 expression was markedly reversed by PGC-1α knockdown (Fig. 6E, F). Conversely, the expression of TFAM was significantly inhibited by DDX3X knockdown followed by the knockdown of NRF-2, but there was no significant effect on the expression of PGC-1α compared to that in response to DDX3X knockdown alone (Fig. 6G, H).

Overall, DDX3X promotes APAP-induced liver injury by inhibiting the PGC1α-NRF2 signaling pathway and thereby negatively regulating TFAM expression.

### DDX3X–PGC1α–NRF2 signaling pathway is disrupted in the liver of AILI patients

Finally, we examined the expression of DDX3X, PGC-1α, and NRF-2 molecules in liver tissues of AILI patients. mRNA and protein levels of PGC-1α and NRF-2 in the liver of AILI patients were remarkably decreased compared to normal controls, in contrast, the expression level of DDX3X was higher in patients with AILI than that in normal subjects (Figure S11A, B). Collectively, these data indicate that DDX3X-mediated PGC-1α–NRF2–TFAM signaling pathway disruption may be involved in patients with AILI.

## Discussion

In this study, we found that DDX3X exerts a pro-injury effect by inhibiting the PGC1α–NRF2–TFAM signaling pathway during the pathogenesis of AILI. APAP overdose resulted in reduced TFAM expression, caused massive hepatocyte necrosis, and further promoted mtDNA release into the peripheral blood. TFAM overexpressing protected against APAP-induced mitochondrial dysfunction in mice. Furthermore, we demonstrated that in a primary hepatocyte model, the reduction in TFAM may be regulated by an increase in DDX3X, which suppresses TFAM expression by suppressing the PGC1α–NRF2 signaling pathway and promotes the pathogenic process of AILI.

TFAM which was initially identified as a transcription factor responsible for mtDNA synthesis, also plays an important role in maintaining mtDNA and mitochondrial integrity. TFAM has been shown to play a core role in mtDNA stress-mediated inflammatory responses (Zhao et al. 2021a). In addition, it has been shown that alterations in TFAM levels or mtDNA copy number are associated with acute renal failure (Zhao et al. 2021b), tumor progression (Li et al. 2022), skeletal muscle atrophy (Kang et al. 2016), ischemic and nonischemic cardiac dysfunction (He et al. 2021), neurodegeneration (Li et al. 2017) and many other diseases. Stiles et al. showed that TFAM mutations could cause neonatal liver failure associated with mtDNA depletion (Al-Hussaini et al. 2014). However, few studies have clarified the mechanisms of action of TFAM in acute liver injury, especially in AILI. In our study, we found that TFAM expression was significantly reduced in the APAP-induced acute liver injury model for the first time. In APAP-induced hepatotoxicity, apoptotic or necrotic hepatocytes may release damage-associated molecular patterns (DAMPs) (Lee et al. 2019). In our study, mtDNA, a major component of DAMPs, was significantly increased in the peripheral blood of AILI patients. In addition, a major feature of AILI is the altered morphology and dysfunction of mitochondria, while mitochondria are basically restored to a homeostatic state after TFAM overexpression.

DDX3X is a member of the DEAD box helicase family, which is involved in RNA transcription, pre-mRNA splicing, RNA export, and translation (Mo et al. 2021). DDX3X is ubiquitously expressed in human tissues and is involved in many biological processes. In our study, we found that DDX3X was markedly increased in AILI, and since both TFAM and DDX3X are associated with mitochondrial homeostasis, we further investigated whether the two interacted in the AILI model. Through bidirectional intervention experiments, we found that DDX3X knockdown promoted TFAM expression, while DDX3X knockdown followed by TFAM knockdown reversed the protective effect of TFAM. In this study, with the prolongation of APAP stimulation time, the expression level of DDX3X increased significantly and reached the peak at 12 h. The expression level at 24 h was lower than that of the 12 h group, but still higher than the normal level, and thus could still inhibit the expression of TFAM. The expression of TFAM increased slightly at 6 h, which may be attributed to the fact that the liver would show a certain stress response when it was stimulated by external stimuli (Villanueva-Paz et al. 2021), which would lead to the transient increase of TFAM expression to cope with the injury. Transient increase in TFAM expression in response to injury. With the prolongation of APAP stimulation, this transient response to injury was no longer effective, and the expression level of TFAM began to decrease significantly with the continuous increase of DDX3X, thus contributing to APAP-induced liver injury which is consistent with existing studies (Bashir and Morgan 2023). In a study by Luo et al. (Luo et al. 2023) it was found that the expression of DDX3X was down-regulated in liver tissues after stimulating mice with 350 mg/kg of APAP for 12 h, whereas hepatic tissue-specific knockdown of DDX3X exacerbated the liver injury caused by APAP. This is different from our findings, and we believe that the causes of this phenomenon may be the following. First, the two studies used different doses of APAP, and different doses of APAP stimulation may lead to different expression trends of the same molecule (Kotulkar et al. 2023). In addition, the study by Luo et al. used mice with hepatocyte-specific knockout of DDX3X, whereas the DDX3X knockout mice used in our study were constructed with siRNA, which knocked out DDX3X in hepatocytes while knocking out DDX3X in other cells such as immune cells (Samir and Kanneganti 2022). When DDX3X was knocked down in hepatocytes, DDX3X in other cells might exert compensatory effects, whereas systemic knockdown failed to exert compensatory effects, resulting in a protective trend after DDX3X knockdown using siRNA.

PGC-1 family members such as PGC-1α and PGC-1β are important transcriptional co-activators associated with mammalian mitochondrial biogenesis and energy metabolism (Piccinin et al. 2019). The coactivator PGC-1α plays a central role in the regulatory network and mediates the transcriptional regulation of mitochondrial biogenesis and respiratory function (Wu et al. 1999). It has been shown that PGC-1α plays a key role in skeletal muscle mitochondrial biogenesis. PGC-1α acts synergistically with NRFs including NRF-1 and NRF-2 to promote the expression of a variety of nuclear-encoded genes and TFAM (Puigserver et al. 1998). In addition, when PGC-1α is introduced into muscle cells, it significantly induces the gene expression of NRF-1, NRF-2, and TFAM (Jornayvaz 2010). Silencing of NRFs produced attenuated TFAM expression and reduced mtDNA levels. Thus, in our study, we explored whether DDX3X further suppressed TFAM expression by inhibiting the expression of PGC-1 or NRFs. We found that the gene and protein levels of NRF-2 and PGC-1α were significantly increased after DDX3X knockdown in the APAP-treated animal and cell model. We then interfered with NRF-2 and PGC-1α in the AILI cell model and found that DDX3X negatively regulates TFAM by inhibiting the PGC1α-NRF2 signaling pathway thereby negatively regulating TFAM expression and promoting APAP-induced liver injury.

There are some limitations in the current study. First, we included only 3 liver tissue specimens from patients with AILI, which is due to the difficulty in obtaining specimens because AILI patients have an acute liver injury with rapid disease progression and few meet the criteria for liver transplantation. Second, we demonstrated the role of TFAM overexpression and DDX3X knockdown in the pathogenesis of AILI, and we could further improve the model of TFAM knockdown and DDX3X overexpression to verify the mechanism of their action in the opposite direction. Finally, we demonstrated the inhibitory effect of DDX3X on NRF-2 and PGC-1α by detecting changes in the levels of these two molecules after DDX3X intervention, but the specific regulatory mechanisms of DDX3X on these two molecules have not been further investigated.

In conclusion, we clarified that during the progression of AILI pathogenesis, DDX3X promotes APAP-induced hepatotoxicity by inhibiting the PGC1α-NRF2 signaling pathway and thereby negatively regulating TFAM expression, which exacerbates hepatocyte necrosis and mitochondrial dysfunction.

## Supplementary Information

Supplementary Material 1.

## Data Availability

No datasets were generated or analysed during the current study.
