# Supplementary material for: Depressed TFAM promotes acetaminophen-induced hepatotoxicity regulated by DDX3X–PGC1α–NRF2 signaling pathway
Source: Mol Med. 2024 Dec 19;30:246. doi: 10.1186/s10020-024-01017-0 (PMC11657569; doi:10.1186/s10020-024-01017-0)
Supplement: Supplementary file 1 — Supplementary Material 1. [file 10020_2024_1017_MOESM1_ESM.pdf]

Supplemental Information

Supplemental Table 1

Demographic and clinical characteristics of the different serum study groups

**Parameters**	**Normal (n=20)**	**AILI (n=40)**	***p* value**
Age(years)	41.7±9.7	44.7±12.2	0.3489
Gender (male/female)	12/8	21/19	
ALT (U/L)	20.3±4.1	5039.4±1852.8	<0.0001
AST (U/L)	18.5±4.6	4064.4±1759.5	<0.0001
Albumin (g/L)	43.4±3.2	28.3±2.6	<0.0001
Total bilirubin (μmol/l)	8.8±2.9	10.3±3.0	0.0662

AILI: Acetaminophen-induced acute liver injury; ALT: Alanine aminotransferase; AST: Aspartate aminotransferase

Supplemental Table 2

List of primers

Genes	Forward Primers (5'-3')	Reverse Primers (5'-3')
Mus-HPRT	AGTCCCAGCGTCGTGATTAG	GCCTCCCATCTCCTTCATGA
Mus-TFAM	CCGAGGTGGTTTTCATCTGT	TCCGCCCTATAAGCATCTTG
mtDNA D-loop	CACAGCCACTTTCCACACAG	TGGTTAGGCTGGTGTTAGGG
Mus-DDX3X	TGGAGGAAGTACAGCAAGCA	TCCCCTTGATCCACTTCCAC
Mus-CXCL1	GCTGGGATTCACCTCAAGAA	TGGGGACACCTTTTAGCATC
Mus-CXCL2	AGTGAACTGCGCTGTCAATG	TTCAGGGTCAAGGCAAACTT
Mus-CXCL10	AAGTGCTGCCGTCATTTTCT	GTGGCAATGATCTCAACACG
Mus-TNFα	CGTCAGCCGATTTGCTATCT	CGGACTCCGCAAAGTCTAAG
Mus-IL4	TCAACCCCCAGCTAGTTGTC	TGTTCTTCGTTGCTGTGAGG
Mus-IL6	AGTTGCCTTCTTGGGACTGA	TCCACGATTTCCCAGAGAAC
Mus-IL10	CCAAGCCTTATCGGAAATGA	TTTTCACAGGGGAGAAATCG
Mus-IL12	AAGGAACAGTGGGTGTCCAG	CATCTTCTTCAGGCGTGTCA
Mus-IL18	ACGTGTTCCAGGACACAACA	ACAAACCCTCCCCACCTAAC
Mus-IL22	CAACTTCCAGCAGCCATACA	GTTGAGCACCTGCTTCATCA
Mus-DDX3X	TGGAGGAAGTACAGCAAGCA	TCCCCTTGATCCACTTCCAC
Mus-PGC1α	AGCCTCTTTGCCCAGATCTT	GGCAATCCGTCTTCATCCAC
Mus-PGC1β	TCTGCCAACGGAAACAAAGG	GCTGCTGTCCTCAAATACGG
Mus-NRF1	GTCCGCACAGAAGAGCAAAA	CTACTGTTGCCCCTGTACCA
Mus-NRF2	ACATGGAGCAAGTTTGGCAG	TGGAGAGGATGCTGCTGAAA

Human-HPRT	CCTGGCGTCGTGATTAGTGA	GCCTCCCATCTCCTTCATCA
Human-TFAM	GTGGGAGCTTCTCACTCTGG	TAGGGCTTTTTCTCCTGCAA

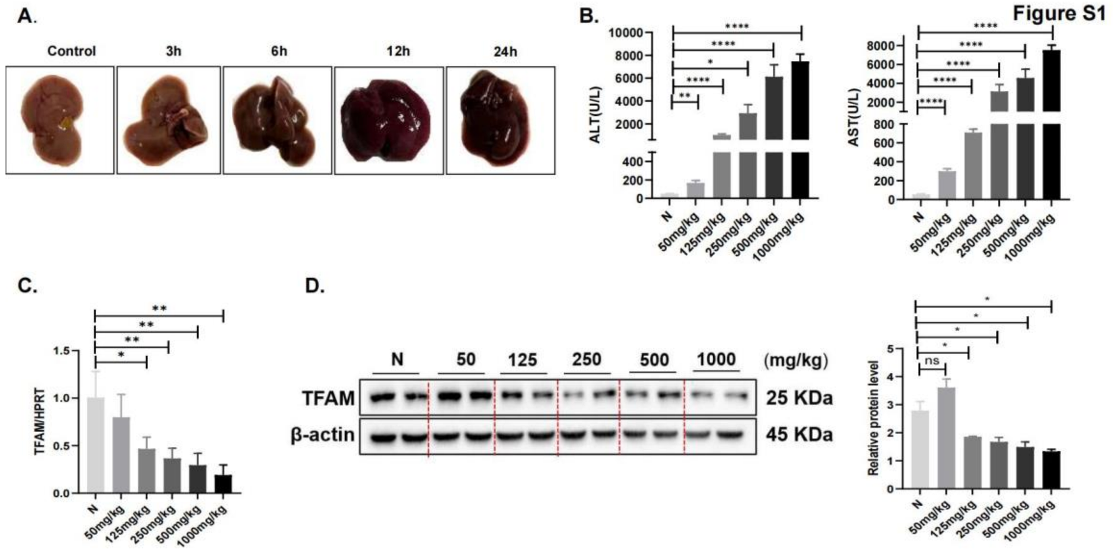

Supplemental Fig. 1. Expression trends of TFAM in liver tissues of AILI Mouse model.

A. Appearance of mouse liver at different times of APAP stimulation. N=6 for each group.

B. Serum ALT and AST levels in mouse models stimulated by different concentrations of APAP solution.

C. Gene expression of TFAM was measured by quantitative real-time PCR in mouse liver tissue samples.

D. Protein levels of TFAM in mouse liver tissue samples were detected by western blotting and quantitated with the Image J software. The blots and images are representative of three independent experiments.

$^{*}p < 0.05$; $^{**}p < 0.01$; $^{***}p < 0.001$; $^{****}p < 0.0001$; ns not significant.

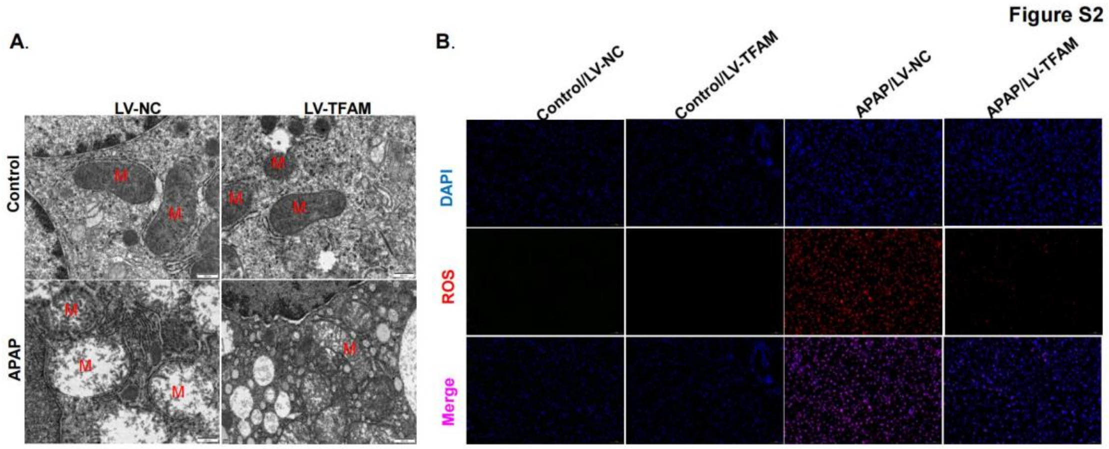

Supplemental Fig. 2. Overexpression of TFAM alleviates APAP-induced mitochondrial dysfunction.

A. Transmission electron microscope observation of mitochondrial morphology (scale bar, 500nm).

B. Immunofluorescence observation of hepatic ROS content in AILI model group and TFAM intervention group (red: ROS-positive cells; blue: DAPI; scale bar, 50 μm).

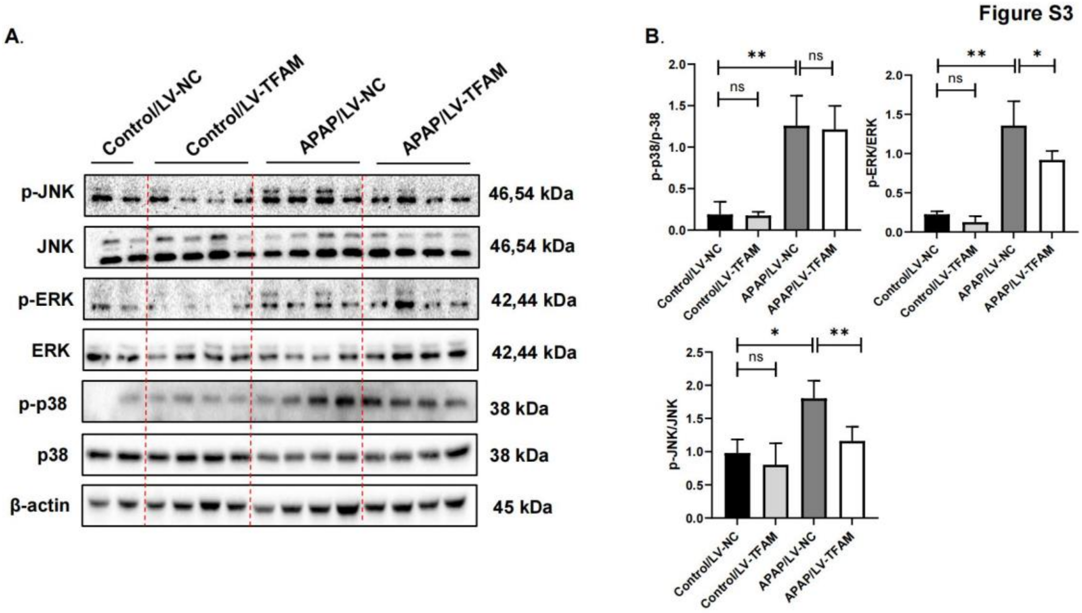

Supplemental Fig. 3. Effect of overexpression of TFAM on MAPK signaling pathway.

A-B. p-JNK, JNK, p-ERK, ERK, p-p38 and p-38 expression levels in liver tissue were measured by western blotting and quantitated with the Image J software. N=6 for each group. The blots and images are representative of three independent experiments.

Figure S4

A.

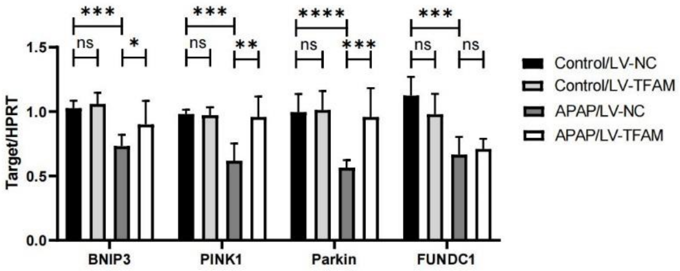

Supplemental Fig.4. Effect of overexpression of TFAM on mitochondrial autophagy-related molecules.

A. Gene expression of mitochondrial autophagy-related molecules was measured by quantitative real-time PCR in mouse liver tissue samples. N=6 for each group.

Figure S5

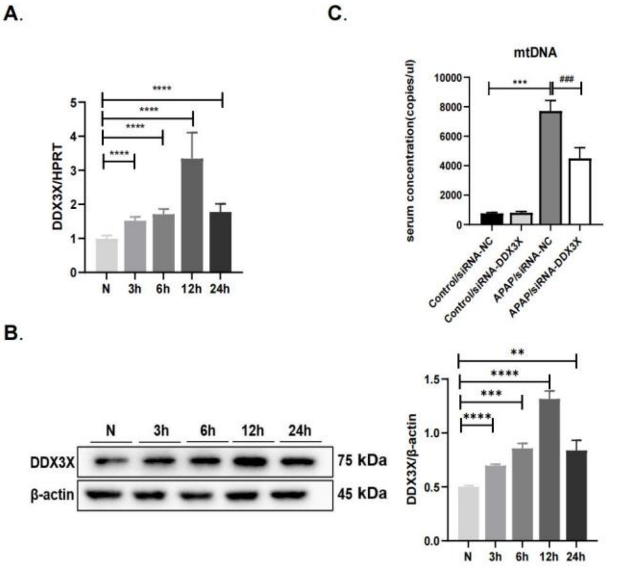

Supplemental Fig. 5. Expression levels of DDX3X in AILI models.

A. Gene expression of DDX3X was measured by quantitative real-time PCR in time-gradient-stimulated primary hepatocytes.

B. Protein levels of DDX3X in time-gradient-stimulated primary hepatocytes were detected by western blotting and quantitated with the Image J software. The blots and images are representative of three independent experiments. Data from 3 independent experiments are shown as mean ± SD.

C. Serum levels of mtDNA in different differently treated mouse models. N=6 for each group.

* p<0.05, ** p<0.01, *** p<0.001, **** p<0.0001; ### p<0.001.

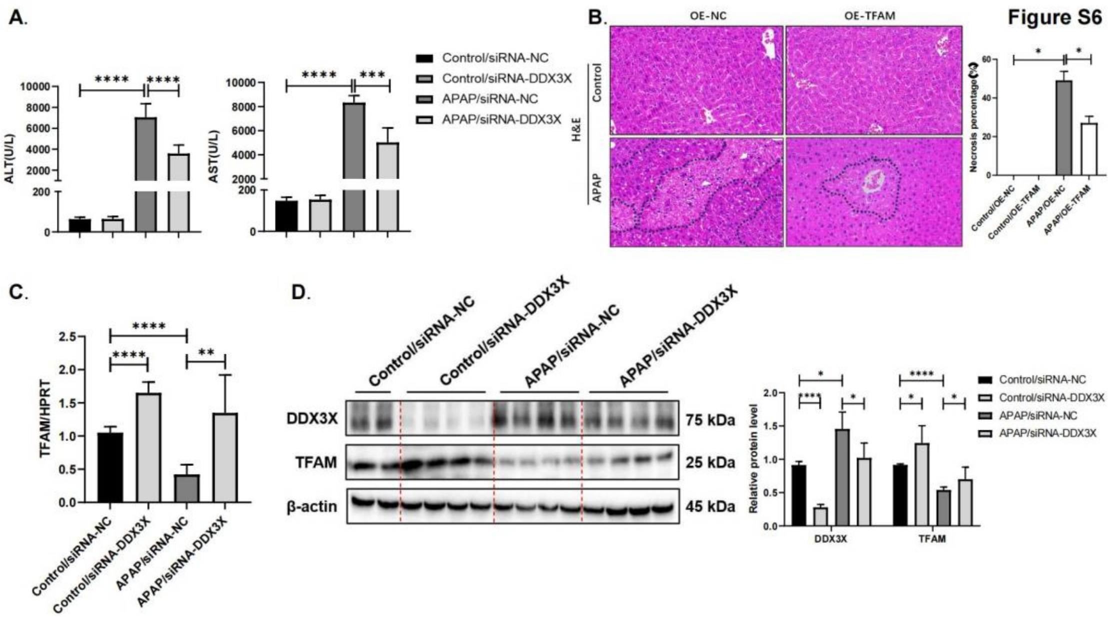

Supplemental Fig. 6. DDX3X promotes APAP-induced liver injury by negatively regulating TFAM in vivo.

A. Detection of the effect of knockdown of DDX3X on serum ALT and AST in control female mice or APAP model female mice. N=6 for each group.

B. Representative images of H&E-stained liver sections. The areas surrounded by a black line and arrow are areas of liver tissue damage (magnification: 200×). Data represent the mean ratio of the necrotic area to the total area ± SD.

C. Gene expression of TFAM was measured by quantitative real-time PCR in female mouse liver tissue from AILI mice with DDX3X of knockdown.

D. Protein levels of TFAM in female mouse liver tissue samples of AILI mice with DDX3X knockdown were detected by western blotting and quantitated with the Image J software. The blots and images are representative of three independent experiments.

Figure S7

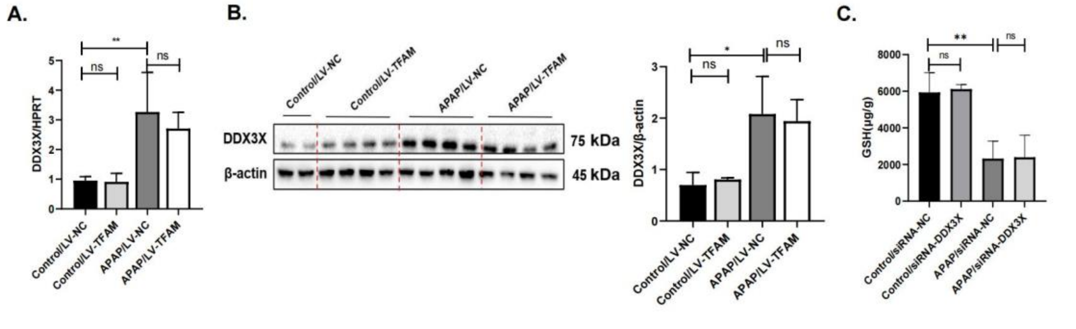

Supplemental Fig. 7. Effect of TFAM intervention on DDX3X in AILI mouse models.

A. Gene expression of DDX3X was measured by quantitative real-time PCR in mouse liver tissue samples of AILI mice overexpressing TFAM. N=6 for each group.

B. Protein levels of DDX3X in mouse liver tissue samples of AILI mice overexpressing TFAM were detected by western blotting and quantitated with the Image J software. The blots and images are representative of three independent experiments.

C. GSH content in liver tissues of different groups.

$^{*}p<0.05$, $^{**}\ p<0.01$, ns not significant.

Figure S8

A.

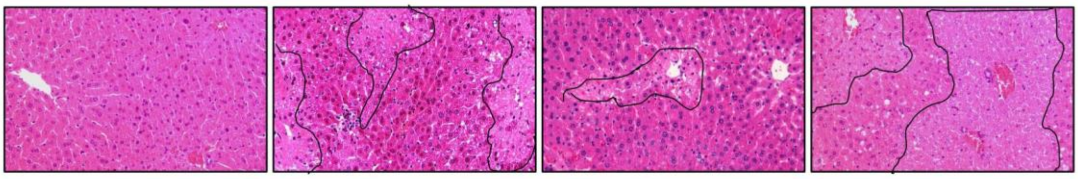

Supplemental Fig. 8. DDX3X promotes APAP-induced liver injury by negatively regulating TFAM in vivo.

A. Representative images of H&E-stained liver sections. The areas surrounded by a black line and arrow are areas of liver tissue damage (magnification: 200×).

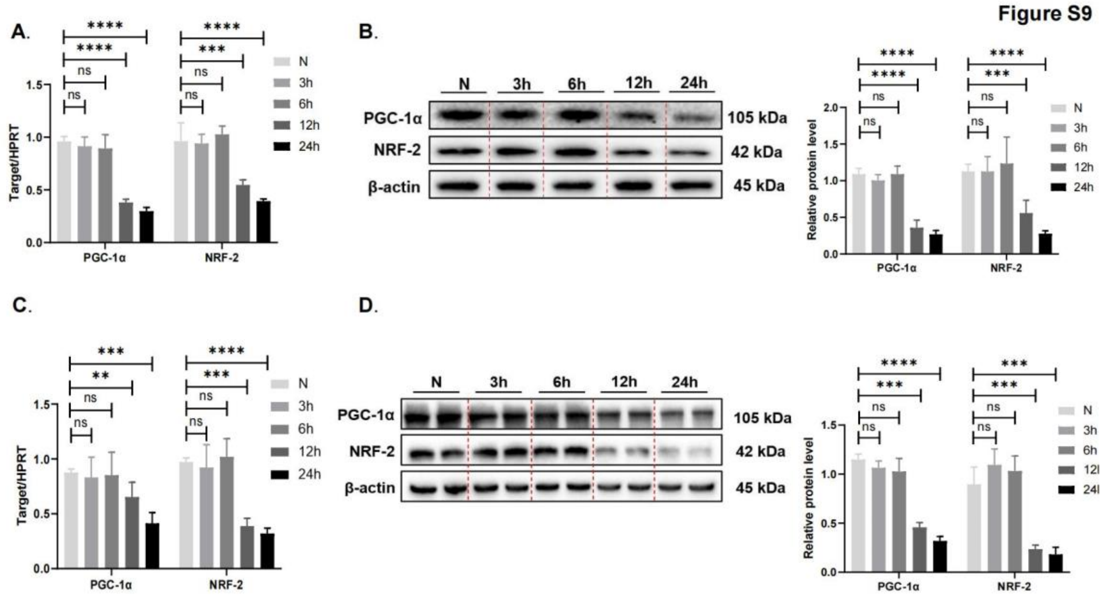

Supplemental Fig. 9. Expression levels of PGC-1α and NRF-2 in AILI animal and cell models.

A. Gene expression of PGC-1α and NRF-2 was measured by quantitative real-time PCR in time-gradient-stimulated primary hepatocytes.

B. Protein levels of PGC-1α and NRF-2 in time-gradient-stimulated primary hepatocytes were detected by western blotting and quantitated with the Image J software. The blots and images are representative of three independent experiments. Data from 3 independent experiments are shown as mean ± SD.

C. Gene expression of PGC-1α and NRF-2 was measured by quantitative real-time PCR in time-gradient-stimulated mouse liver tissues.

D. Protein levels of PGC-1α and NRF-2 in time-gradient-stimulated mouse liver tissues were detected by western blotting and quantitated with the Image J software. The blots and images are representative of three independent experiments. Data from 3 independent experiments are shown as mean ± SD.

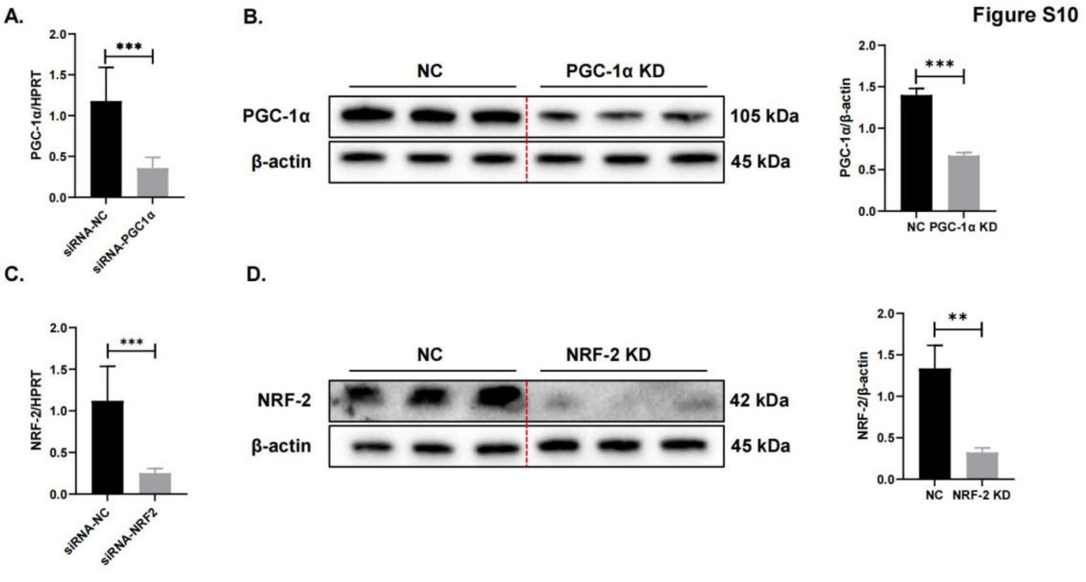

Supplemental Fig. 10. The efficiency of target siRNAs was validated.

A. Primary hepatocytes were treated with PGC-1α siRNA oligos for 24h, and the expression level of targets was measured by quantitative real-time PCR.

B. Primary hepatocytes were treated with PGC-1α siRNA oligos for 24h, and the expression level of targets was measured by WB. Data from 3 independent experiments are shown as mean ± SD.

C. Primary hepatocytes were treated with NRF-2 siRNA oligos for 24h, and the expression level of targets was measured by quantitative real-time PCR.

D. Primary hepatocytes were treated with NRF-2 siRNA oligos for 24h, and the expression level of targets was measured by WB. Data from 3 independent experiments are shown as mean ± SD.

** p<0.01, *** p<0.001.

Figure S11

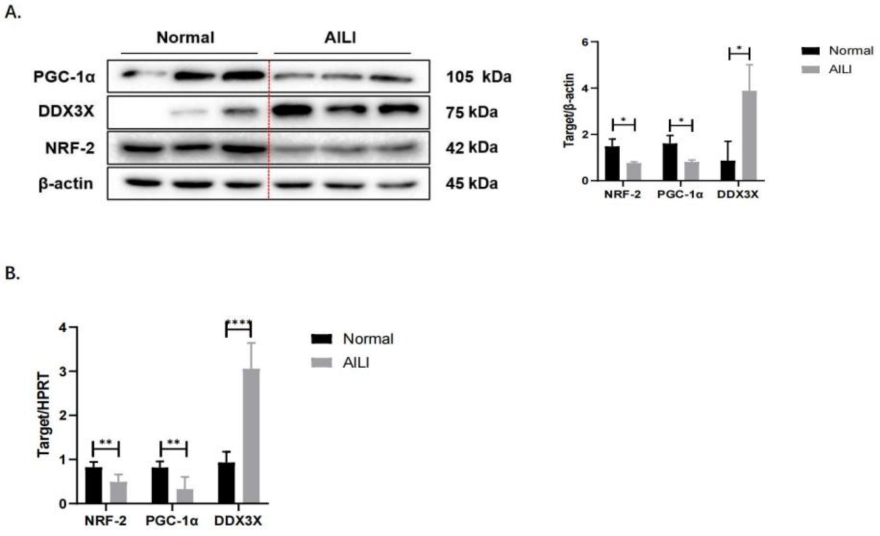

Supplemental Fig. 11. DDX3X- PGC1α-NRF2 signaling pathway is disrupted in the liver of AILI patients.

A. Protein levels of DDX3X, PGC-1α and NRF-2 in human liver tissue samples were detected by western blotting and quantitated with the Image J software. N=3 for each group.

B. Gene expression of DDX3X, PGC-1α and NRF-2 were measured by quantitative real-time PCR in normal subjects and AILI patients. N=3 for each group.

$^{*}p < 0.05$; $^{**}p < 0.01$; $^{***}p < 0.001$; $^{****}p < 0.0001$.
